# Sodium Glucose Cotransporter 2 Inhibitors Reduce the Risk of Heart Failure Hospitalization in Patients With Type 2 Diabetes Mellitus: A Systematic Review and Meta-Analysis of Randomized Controlled Trials

**DOI:** 10.3389/fendo.2020.604250

**Published:** 2021-01-15

**Authors:** Ailing Zhang, Xufei Luo, Haiyang Meng, Jian Kang, Guijun Qin, Yaolong Chen, Xiaojian Zhang

**Affiliations:** ^1^ Department of Pharmacy, The First Affiliated Hospital of Zhengzhou University, Zhengzhou, China; ^2^ School of Public Health, Lanzhou University, Lanzhou, China; ^3^ Department of Endocrinology, The First Affiliated Hospital of Zhengzhou University, Zhengzhou, China; ^4^ Institute of Health Data Science, Lanzhou University, Lanzhou, China; ^5^ Evidence-based Medicine Center, School of Basic Medical Sciences, Lanzhou University, Lanzhou, China; ^6^ Lanzhou University, an Affiliate of the Cochrane China Network, Lanzhou, China; ^7^ Chinese GRADE Center, Lanzhou, China; ^8^ Key Laboratory of Evidence Based Medicine and Knowledge Translation of Gansu Province, Lanzhou University, Lanzhou, China

**Keywords:** sodium glucose cotransporter 2 inhibitors, type 2 diabetes mellitus, heart failure hospitalization, systematic review, meta-analysis

## Abstract

**Aim:**

To evaluate the impact of sodium glucose cotransporter 2 inhibitors (SGLT-2i) on risk of heart failure hospitalization in patients with type 2 diabetes.

**Methods:**

We searched the PubMed, Embase, The Cochrane Library, CNKI, Wanfang, CBM, and other web knowledge databases for data from randomized controlled trials. We performed statistical analyses by using review Manager (RevMan) 5.3 and STATA 12.0 for meta-analysis.

**Results:**

Eight randomized controlled trials that compared SGLT-2i *versus* placebo met our inclusion criteria and were included in the study. The final meta-analysis included a total of 55,763 type 2 diabetes patients. Compared with placebo, SGLT-2i reduced the risk of heart failure hospitalization (RR, 0.63; 95% CI, 0.53 to 0.74; *P* < 0.00001), MACE (defined as cardiovascular death, myocardial infarction, or ischemic stroke) (RR, 0.92; 95% CI, 0.86 to 0.98; *P* < 0.007), cardiovascular death (RR, 0.78; 95%CI, 0.62 to 0.99; *P* = 0.04) in type 2 diabetes patients. SGLT-2i could reduce the risk of death from any cause (RR, 0.77; 95% CI, 0.59 to 1.01; *P* = 0.06) without statistical significance in type 2 diabetes patients.

**Conclusion:**

Compared with placebo, SGLT-2i may reduce the risk of heart failure hospitalization, MACE, and cardiovascular death. Therefore, SGLT-2i may be an ideal choice for type 2 diabetes mellitus patient with heart failure. These results will help inform practitioners, patients, and authorities making appropriate choices in hypoglycemic therapy clinical practice.

## Introduction

Diabetes mellitus is one of the most important independent risk factor for cardiovascular diseases (1). Compared with non-diabetic individuals, diabetes confers about a two-fold excess risk for a wide range of cardiovascular diseases, independently from other conventional risk factors (2). Recent evidence suggests that heart failure is another predominant cardiovascular disease, which is as common as ischemic events in type 2 diabetes patients (3). Besides, heart failure hospitalization is a significant burden of cardiovascular death among type 2 diabetes mellitus patients (4). Patients with type 2 diabetes mellitus and concomitant heart failure require complex medication regimens; what’s more, glucose-lowering medications such as thiazolidinedions (5), dipeptidyl peptidase-4 (DPP-4) inhibitors (6, 7) (saxagliptin and alogliptin) also can increase risk for heart failure hospitalization. Therefore, the cardiovascular safety benefits of glucose-lowering agents could be an ideal choice for type 2 diabetes patients with heart failure.

Recent clinical trials involving the SGLT-2i, such as empagliflozin (8), canagliflozin (9), dapagliflozin (10), and ertugliflozin (11) have surprisingly demonstrated a reduction in the risk of heart failure outcomes among patients with type 2 diabetes who have established cardiovascular disease (CVD) or are at high cardiovascular failure risk. Although three published meta-analyses have reported the discovery of SGLT-2i reducing heart failure hospitalization with only three RCTs; five other RCTs have been published after these meta-analyses published. Therefore, it is necessary to update the meta-analysis. So we collated the most recent evidence into the prior information, and aim to identify the impact of SGLT-2i on heart failure hospitalization in patients with type 2 diabetes.

## Methods

### Data Sources and Search Strategy

We conducted a computerized literature search of PubMed, EMbase, the Cochrane Library, CNKI, Wanfang, CBM, and other data resources (we search for articles published up to November 19, 2020) by using the Boolean combinations of the key terms “Sodium-Glucose Transporter 2 Inhibitors” AND “Diabetes Mellitus” AND “Heart Failure.” The Medical Subject Headings (MeSH) or keywords were used when the searching database with option was available. Language of the published papers was restricted to English and Chinese, and only human studies were included. Besides, references of the included articles were examined to identify additional studies.

### Study Selection

Two authors independently screened the studies and extracted data by using a standardized form. All disagreements were resolved with group consensus. To conclude, the inclusion criteria were as follows: (1) Type 2 diabetes patients were treated with SGLT-2i; (2) Data on re-hospitalization due to heart failure were well documented in the literature; (3) The research follow-up ≥1 year with complete data records; (4) The research was a randomized controlled trail; (5) Language of literature were English or Chinese. Studies were excluded from the primary analysis if any of the following was met: (1) Letters, case reports, review articles, conference abstracts, editorials, insufficient information researches, and duplicate studies. (2) The mechanism of SGLT2 for heart failure, animal experiment, test protocol was reported without relevant study results.

### Data Extraction and Quality Assessment

The following information was extracted from each included study: the first author’s last name, year of publication, geographic origin, study design, age and gender of patients, type 2 diabetes duration, glycosylated hemoglobin level, follow-up period, sodium-glucose transporter 2 inhibitors regimens, total number of participants in clinical trials. The primary outcome was heart failure hospitalization and the key secondary outcomes were MACE, cardiovascular death, and death from any cause.

The methodological quality of the included studies was evaluated separately by two authors using Cochrane risk of bias tool (12), with consensus reached when different opinions exist on the results. The Cochrane risk of bias tool was used to assess the quality of the RCTs based on five domains: random sequence generation (selection bias), allocation concealment (selection bias), blinding (performance bias and detection bias), incomplete outcome data (attrition bias), and selective reporting (reporting bias). Two reviewers assessed the quality of the evidence independently using the Grading of Recommendations Assessment, Development and Evaluation (GRADE) approach (13, 14). For evidence body of RCT, the quality can be downgraded for five reasons (study limitations, consistency of effect, imprecision, indirectness, and publication bias). The quality of the evidence will be graded as high, moderate, low, and very low.

### Statistical Analysis

Rigorous statistical analysis was performed using Review Manager (RevMan) program version 5.3 (Cochrane Collaboration, Oxford, UK) for meta-analysis and STATA statistical software (Version 12.0, Stata Corporation, College Station, TX, USA) for sensitivity analyses and Egger’s linear regression test. The Risk Ratio (RR) with corresponding 95% confidence interval (95% CI) were calculated to evaluate the association between sodium-glucose transporter 2 inhibitors and heart failure hospitalization, cardiovascular death, and death from any cause. The Z test was used to estimate the statistical significance of pooled RRs. Between-study heterogeneity was assessed by Cochran’s *Q*-statistic and *I* ^2^ test (15). If the *Q*-test exhibited a *P* > 0.05 or the *I* ^2^ test showed <50%, which means that these studies were homogeneous, the fixed-effects model was conducted; otherwise, the random-effect model was used. We also use sensitivity analyses to explore sources of heterogeneity and *P* < 0.05 was considered as the signal of existence of the statistical significance heterogeneity. Funnel plots and Egger’s linear regression test was used to investigate the potential publication bias (16).

## Results

### Study Characteristics and Selection

**Figure 1:**
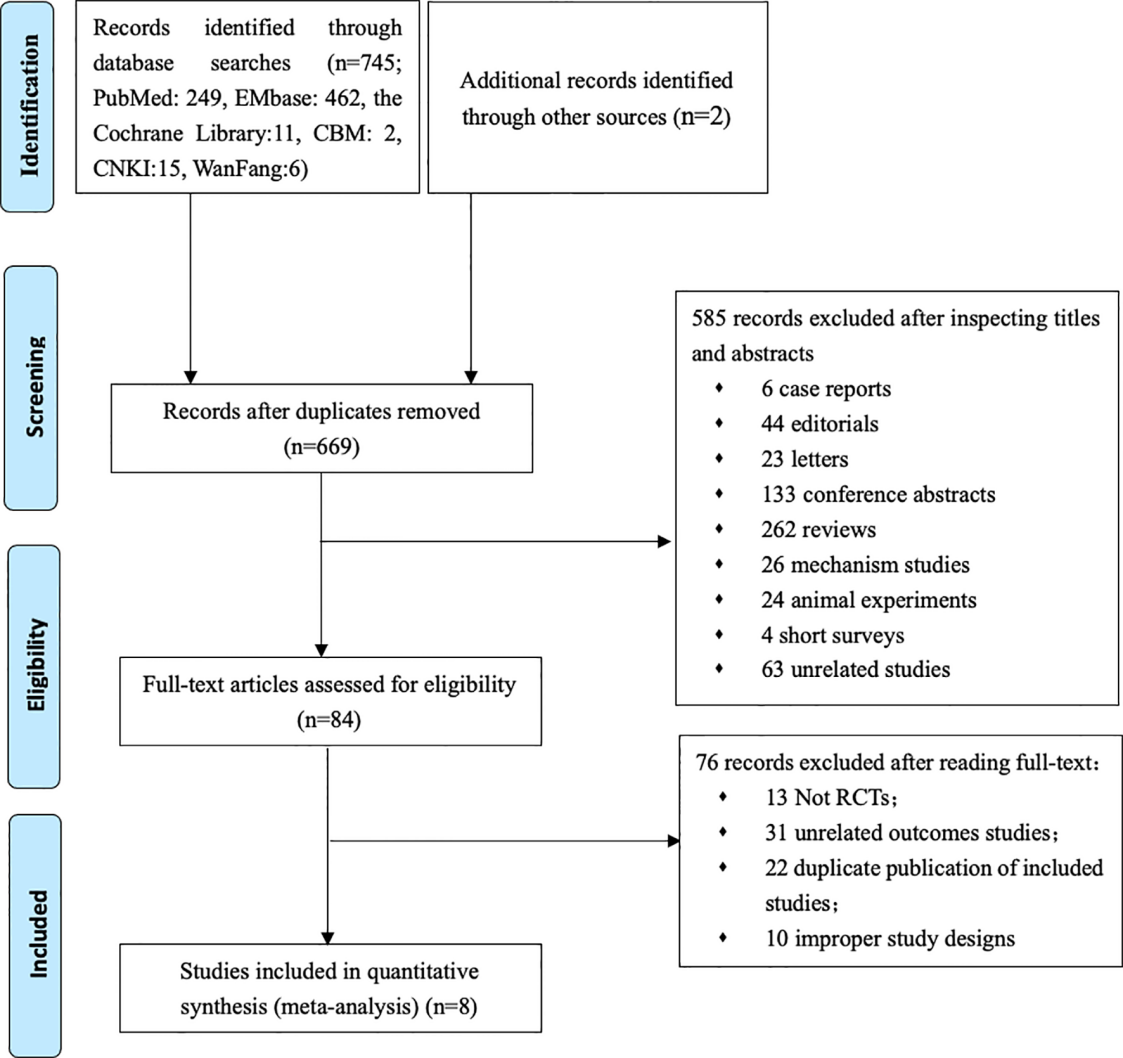
Flow chart of study selection.

Our primary searches originally yielded 747 articles, 669 papers remaining after the removal of duplicates. After reading through titles and abstracts, 6 case reports, 44 editorials, 23 letters, 133 conference papers, 262 reviews, 26 mechanism studies, 24 animal experiments, 4 short surveys, 63 unrelated studies were excluded; 84 studies remained. We reviewed the full texts of the remaining articles, and furtherly excluded 13 non-RCT studies, 31 literatures with irrelevant outcomes, 22 overlapping data with the included study, 10 literatures on pilot programed design. Finally, 8 remaining studies were included in the meta-analysis (**Figure 1**) (8–11, 17–20). Characteristics of the included trials are summarized in **Table 1**. In total, 55,763 type 2 diabetes mellitus patients were randomly assigned to receive different SGLT-2i (empagliflozin, canagliflozin, dapagliflozin) and placebo. Participants were generally middle-aged (mean age 63.0–66.4). Follow-up duration ranged from 1 to 4.2 years.

**Table 1:** Demographic and clinical characteristics of studies included in the meta-analysis.

Author	Year	Location	Study design	Patients (female%)	Age(mean)	T2DM duration(year)	Complication	HbA1c(%)	Follow-up (year)	Interventions	Control measure	Dose(mg/d)	Sample size(Interventions/control)
Zinman (8)	2016	United States, Argentina, Australia, Belgium, etc. 615 centers of 40 countries	RCT	2,036 (29%)	63.1	>10	Myocardial infarction, coronary artery disease, coronary revascularization coronary artery	7.0–10	3.1	*empagliflozin*	placebo	10/25	7,020 (4,687/2,333)
Radholm (9)	2018	United States, Argentina, Australia, Belgium, etc. 313 centers of 26 countries	RCT	3,631 (35.8%)	63.3	13.5	Hypertension, atrial fibrillation	7.0–10.5	3.6	*canagliflozin*	placebo	100/300	10,142 (5,795/4,347)
McMurray (10)	2019	UK., United States, Denmark, Germany, etc. 417 centers of 19 countries	RCT	1,109 (23.4%)	66.4	—	Ischemic, nonischemic, atrial fibrillation,	>6.5	1.5	*dapagliflozin*	placebo	10	4,744 (2,373/2,371)
Cannon (11)	2020	United States	RCT	2,471 (30%)	64.4	12.9	Coronary artery disease, heart failure, myocardial infarction, coronary revascularization	7.0–10.5	3.5	*Ertugliflozin*	placebo	5/15	8,238(5,499/2,747)
Wiviott (17)	2019	United States, Argentina, Australia, Belgium, etc. 804 centers of 33 countries	RCT	6,422 (37.4%)	64	11	Coronary artery disease, peripheral artery disease	6.5–12	4.2	*dapagliflozin*	placebo	10	17,160 (8,582/8,578)
Kosiborod (18)	2017	United States	RCT	119 (37.2%)	64.3	13.5–14	Coronary artery disease, atrial fibrillation	7.3–9.1	1	*dapagliflozin*	placebo	10	320 (171/149)
Isreb (19)	2019	United States, Argentina, Australia, Brazil, etc. 593 centers of 34 countries.	RCT	1,492 (33.9%)	63	—	Hypertension, cardiovascular disease	6.5–10.5	2.62	*canagliflozin*	placebo	100	4,401 (2,202/2,199)
Packer (20)	2020	United States, Argentina, Australia, Belgium, etc. 520 centers of 20 countries.	RCT	893 (23.9%)	66.9	—	Heart failure, atrial fibrillation, hypertension	—	1.33	*empagliflozin*	placebo	10	3,730 (1,863/1,867)

### Effect of SGLT-2i on Heart Failure Hospitalization in Patients With Type 2 Diabetes

**Figure 2:**
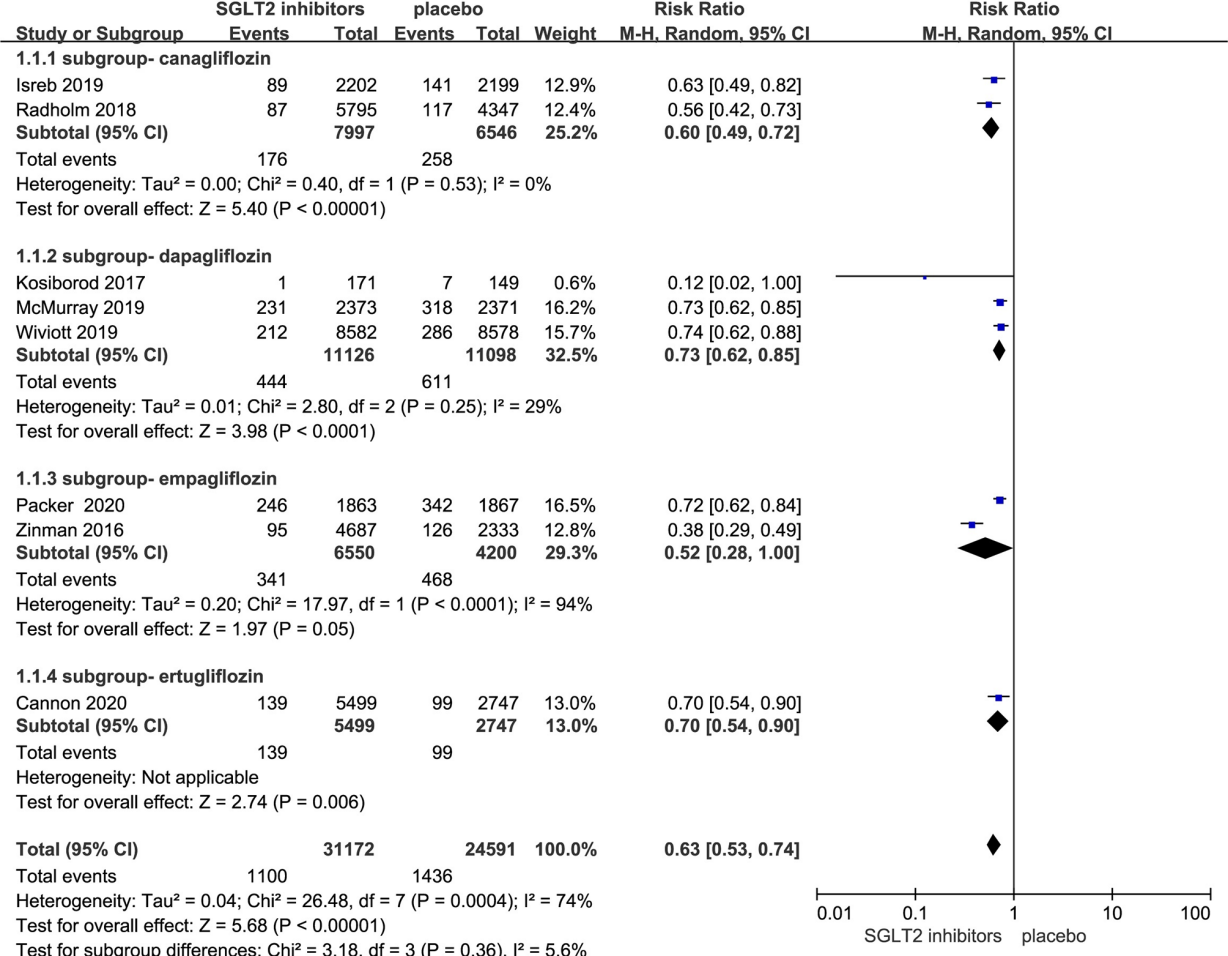
Heart failure hospitalization in type 2 diabetes patients receiving SGLT2 inhibitors versus control.

Eight RCTs evaluating SGLT-2i effect on heart failure hospitalization were identified (8–11, 17–20), 55,763 type 2 diabetes mellitus patients were randomly assigned to 31,172 receive SGLT-2i and 24,591 receive placebo, 1,100 and 1,436 patients were re-hospitalized due to heart failure, respectively. There was significant heterogeneity among eight studies (*P*<0.05, *I* ^2^ = 74%). The meta-analysis using the random-effects model showed that SGLT-2i significantly decreased heart failure hospitalization occurrence in patients with type 2 diabetes compared with control group (RR, 0.63; 95% CI, 0.53 to 0.74; *P* < 0.00001) (**Figure 2**). Subgroup analyses by different agents of SGLT-2i on heart failure hospitalization showed that compared with controls, canagliflozin (RR, 0.60; 95% CI, 0.49 to 0.72; *P* < 0.00001), dapagliflozin (RR, 0.73; 95% CI 0.62 to 0.85; *P* < 0.0001), empagliflozin (RR, 0.52; 95% CI, 0.28 to 1.00; *P* = 0.05) and ertugliflozin (RR, 0.70; 95% CI, 0.54 to 0.90; *P* = 0.006) all significantly decreased the occurrence of heart failure hospitalization in patients with type 2 diabetes. Therefore, the quality of evidence will be graded as high.

### Effect of SGLT-2i on MACE in Patients With Type 2 Diabetes

**Figure 3:**
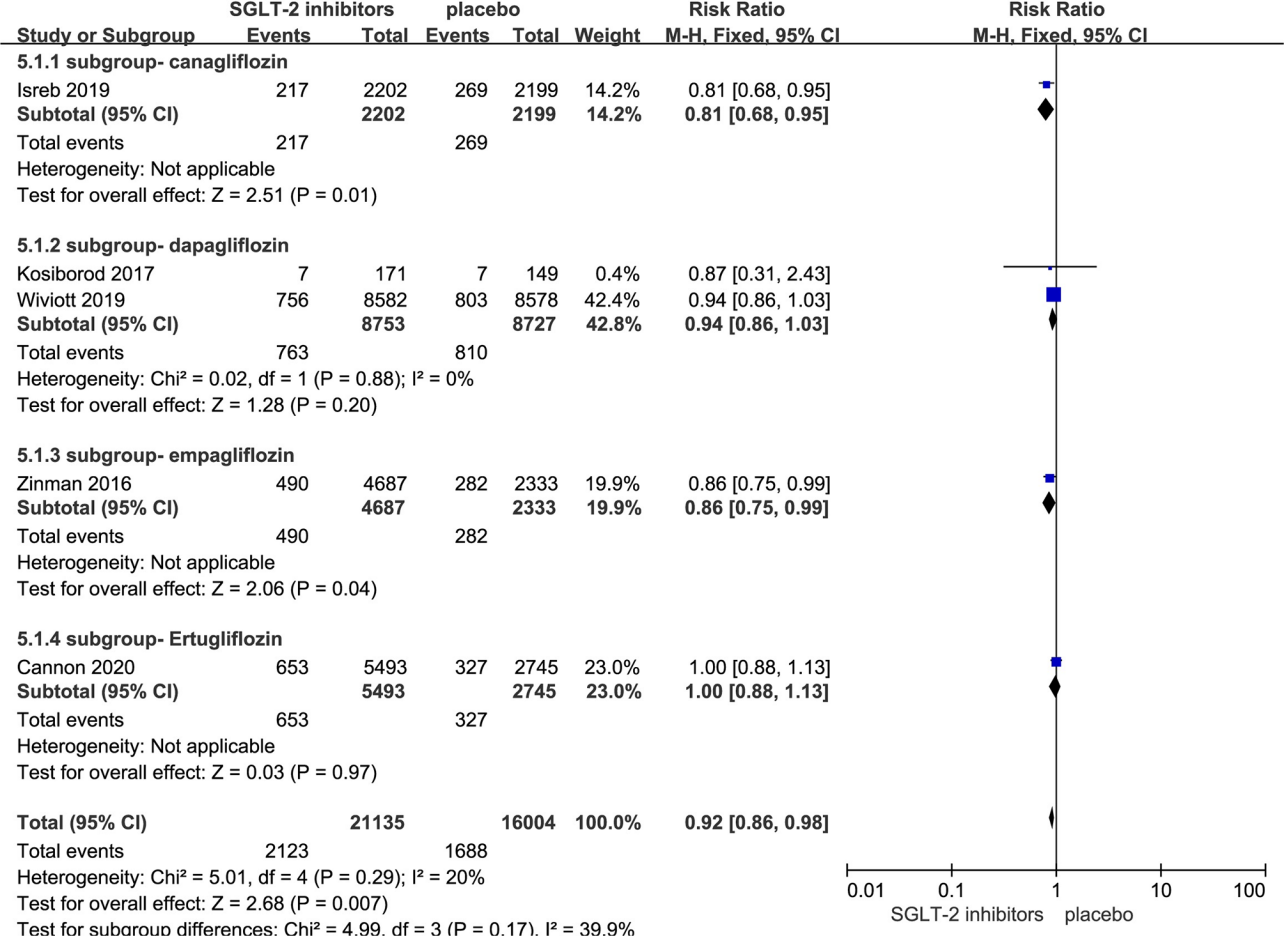
MACE in type 2 diabetes patients receiving SGLT2 inhibitors versus control.

Five RCTs evaluating SGLT-2i on MACE were identified (8, 11, 17–19), 37,139 type 2 diabetes mellitus patients were randomly assigned to 21,135 receive different SGLT-2i and 16,004 receive placebo; 2,123 and 1,688 patients incurred MACE, respectively. There was no significant heterogeneity among five studies (*P *< 0.05, *I* ^2^ = 20%). The meta-analysis using the fixed-effects model showed that SGLT-2i resulted in a lower rate of MACE (RR, 0.92; 95% CI, 0.86 to 0.98; *P* < 0.007) (**Figure 3**). Besides, Subgroup analyses by different agents of SGLT-2i on MACE showed that compared with controls, only canagliflozin (RR, 0.81; 95% CI, 0.68 to 0.95; *P* < 0.01) and empagliflozin (RR, 0.86; 95% CI, 0.75 to 0.99; *P* < 0.04) significantly decreased MACE occurrence in patients with type 2 diabetes. Because of subgroup analyses result, the quality of evidence will be graded as moderate.

### Effect of SGLT-2i on Cardiovascular Death in Patients With Type 2 Diabetes

**Figure 4:**
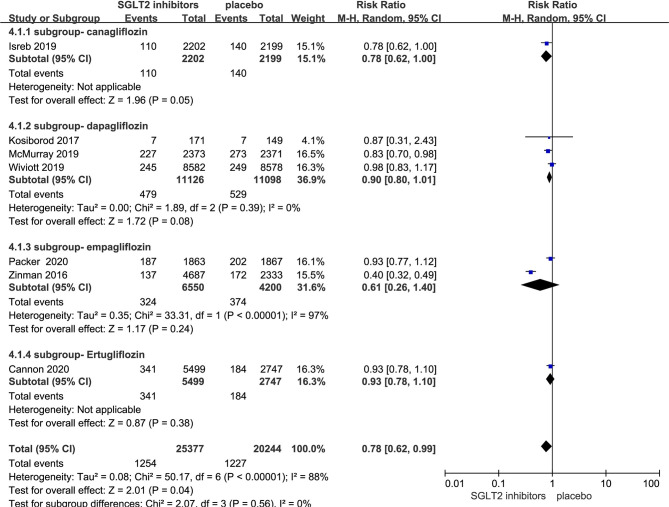
Cardiovascular death in type 2 diabetes patients receiving SGLT2 inhibitors versus control.

Seven RCTs evaluating SGLT-2i on cardiovascular death were identified (8, 10, 11, 17–20), 45,621 type 2 diabetes mellitus patients were randomly assigned to 25,377 receive SGLT-2i and 20,244 receive placebo; 1,254 and 1,227 patients incurred cardiovascular death, respectively. There was significant heterogeneity among seven studies (*P* < 0.05, *I* ^2^ = 88%). The meta-analysis using the random-effects model showed that SGLT-2i could decrease the occurrence of cardiovascular death (RR, 0.78; 95% CI, 0.62 to 0.99; *P* = 0.04) (**Figure 4**). Subgroup analyses by different agents of SGLT-2i on cardiovascular death showed that compared with controls, only canagliflozin decreased cardiovascular death occurrence but without statistical significance in patients with type 2 diabetes (RR, 0.78; 95% CI, 0.62 to 1.00; *P* = 0.05). Because of the significant heterogeneity among seven studies and subgroup analyses result, the quality of evidence will be graded as low.

### Effect of SGLT-2i on Death From Any Cause in Patients With Type 2 Diabetes

**Figure 5:**
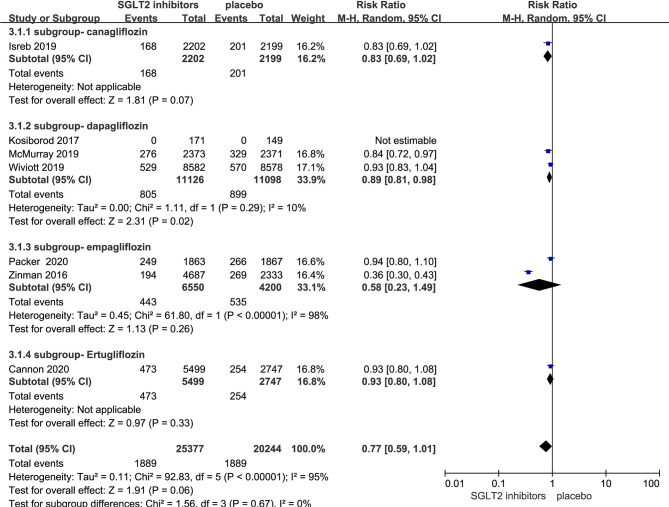
Death from any cause in type 2 diabetes patients receiving SGLT2 inhibitors versus control.

Seven RCTs evaluating SGLT-2i on death from any cause were identified (8, 10, 11, 17–20), 45,621 type 2 diabetes mellitus patients were randomly assigned to 25,377 receive different SGLT-2i and 20,244 receive placebo; 1,889 and 1,889 patients incurred death from any cause, respectively. There was significant heterogeneity among seven studies (*P* < 0.05, *I* ^2^ = 95%). The meta-analysis using the random-effects model showed that SGLT-2i could decrease death occurrence from any cause but without statistical significance (RR, 0.77; 95% CI, 0.59 to 1.01; *P* = 0.06) (**Figure 5**). Subgroup analyses by different agents of SGLT-2i on death from any cause showed that compared with controls, only dapagliflozin (RR, 0.89; 95% CI, 0.81 to 0.98; *P* = 0.02) significantly decreased the occurrence death from any cause in patients with type 2 diabetes. Because of the significant heterogeneity among seven studies and subgroup analyses result, the quality of evidence will be graded as moderate.

### Sensitivity Analysis and Risk of Bias

**Figure 6:**
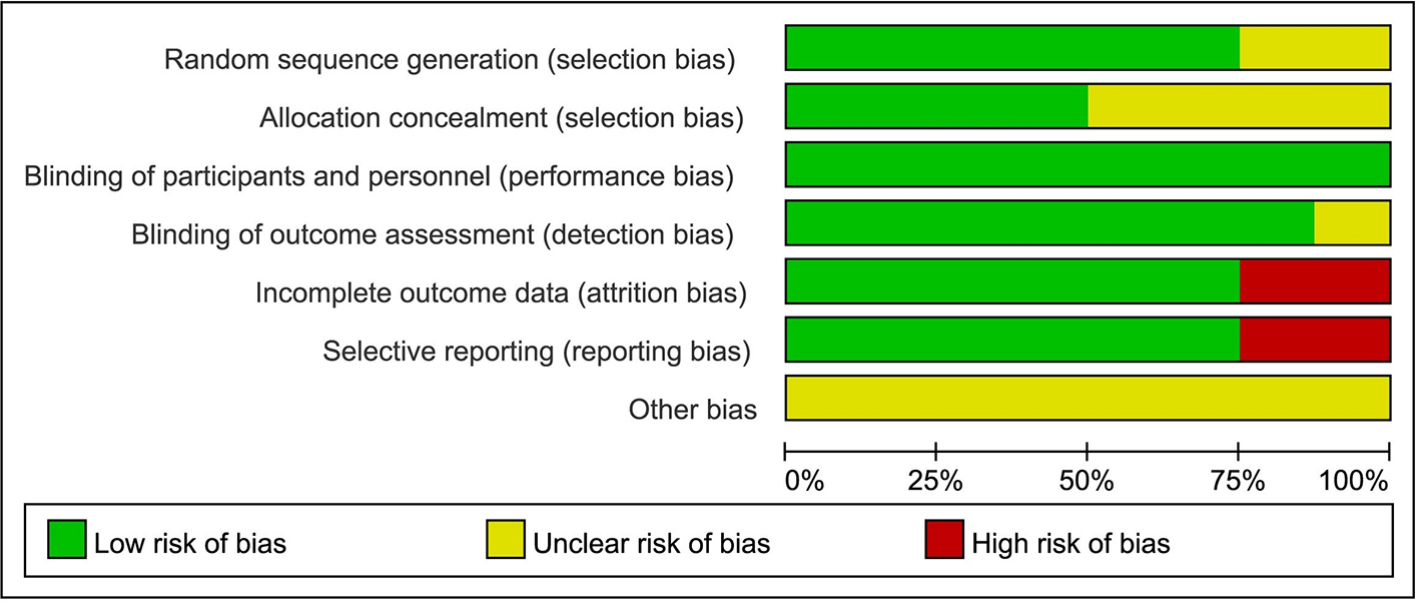
Quality of including studies in the meta-analysis.

Generally, the studies were of high methodologic quality. The overall quality assessment indicated that about one-third had unclear reporting of randomization sequence, one-second had unclear reporting of allocation concealment, one-fourth had unclear reporting of blinding of outcome assessment, and one-third had high risk bias of incomplete outcome data or selective reporting (**Figure 6**). No indication of publication bias was detected with visual assessment of Egger’s test (t = −2.05, p>|t| = 0.087>0.05) in the meta-analysis.

**Figure 7:**
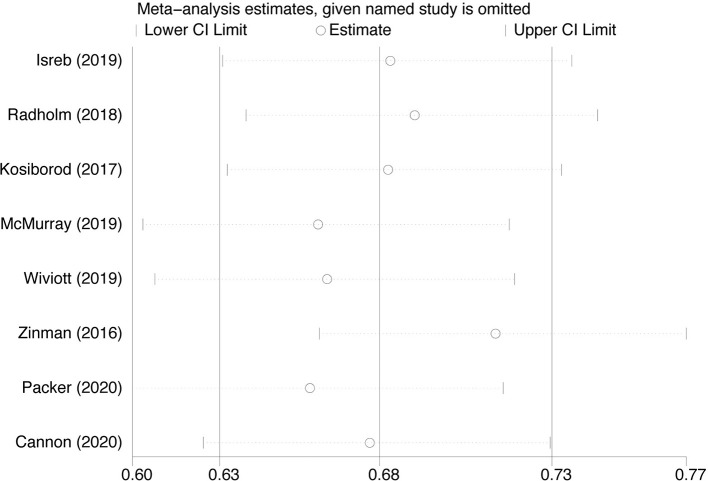
Sensitivity analysis for Heart failure hospitalization in type 2 diabetes patients receiving SGLT2 inhibitors versus control.

Because of significant heterogeneity among the SGLT-2i on heart failure hospitalization studies, we conducted leave-one-out sensitivity analyses. Individual studies were consecutively excluded from the sensitivity analysis to investigate whether the obtained results were robust. The analysis showed that the results obtained in the meta-analysis were statistically robust because the corresponding combined RRs in all of the separate subgroup analyses were relatively stable if any individual study is deleted (**Figure 7**).

## Discussion

Our meta-analysis has shown that SGLT-2i could reduce the risk of heart failure hospitalization and subgroup analyses showed the same result in cases using different agents of SGLT-2i, suggesting that SGLT-2i will be an ideal choice for type 2 diabetes mellitus patient with heart failure. However, SGLT-2i did not result in a lower rate on death from any cause; what’s more, the results were different from the current researches (21, 22). Therefore, these data will help inform practitioners, patients, and authorities regarding the heart failure hospitalization of SGLT-2i in routine clinical practice.

SGLT-2i have gaining more attention, not only for the glycemic benefits but also for favorable effects on cardiovascular outcomes. As we all know, SGLT-2i have been recommended as a first choice in add-on treatment for type 2 diabetes mellitus patient with cardiovascular disease in ADA guidelines (23). The reduction in heart failure hospitalization was also observed in all agents of SGLT-2i in our research. Each study of different agents of SGLT-2i was performed in the different study design with varied backgrounds; so there is significant heterogeneity among the four agents of SGLT-2i on heart failure hospitalization studies. Therefore, we conducted leave-one-out sensitivity analyses and the results were relatively stable if any individual study is deleted. What’s more, the result was largely influenced by dapagliflozin and Empagliflozin, which accounted for 61.8% of the total weight in the analysis. Regardless of the presence or absence of diabetes, dapagliflozin and Empagliflozin reduces the risk of worsening heart failure (10, 20). Dapagliflozin could also prevent hospitalization due to heart failure, regardless of a history of atherosclerotic cardiovascular disease or failure (18). Besides, SGLT-2i also could reduce the risk of MACE and cardiovascular death. Therefore, SGLT-2i will be an ideal choice for type 2 diabetes mellitus patient with heart failure. However, in our meta-analysis, there is concern that SGLT-2i did not result in a lower rate of death from any cause than placebo. Among the four agents of SGLT-2i, only dapagliflozin resulted in a significantly lower risk death from any cause. Given the situation that a relatively small number of current clinical randomized controlled studies were needed, major clinical studies that are currently ongoing will hopefully provide more insight and rigorous data.

The detection of heart failure hospitalization benefit attributed to SGLT-2i was a major discovery. Therefore, several theories of the mechanism of SGLT-2i acting on the heart failure have been postulated (24). Firstly, SGLT-2i could convert cardiovascular risk factors, such as improving metabolic patient profile, capable of including reductions in plasma glucose, blood pressure, body weight, and modifications to the lipid profile (25, 26). Secondly, SGLT-2i induce their diuretic effect by reducing interstitial volume to a much greater extent than intravascular volume which is key to the prevention of fluid overload and heart failure exacerbation leading to hospitalization (27). Thirdly, SGLT-2i could produce a hemodynamic response elicited which creates a favorable environment to reduce cardiac hydrostatic pressure to induce ventricular remodeling and hypertrophy (28, 29). Fourthly, SGLT-2i have been shown to inhibit Na+/H+ exchanger 1 on cardiomyocytes which resulted in increased intracellular sodium and calcium, inducing a pro-oxidant and pro-thrombotic state (30). Fifthly, SGLT-2i induce a reduction in epicardial fat and thus providing another possible mechanism for the beneficial heart failure (31, 32). Sixthly, SGLT-2i could protect renal function by multifactorial, involving electrolyte, vascular, and hydrostatic alterations to increases natriuretic and volume contraction (33). This in turn reduces atrial natriuretic peptide causing vasoconstriction of the afferent renal arterioles. Preservation of renal function is of particular importance for patients with heart failure to avoid volume overload and diuretic resistance (27). Finally, SGLT-2i also could result in erythropoiesis and improve oxygen delivery to tissues (34) and modulate inflammatory and oxidative mediators such as leptin, interleukins (IL), tumor necrosis factor α, cyclooxygenase 2 (COX-2) to decrease the frequency of HF-related hospitalizations (35). Therefore, as described above, the beneficial effects of SGLT2i and their effect on heart failure are multidimensional. In all, SGLT2i could significantly improve heart failure-related outcomes and decrease the frequency of heart failure–related hospitalization.

Our study has several strengths. First, in addition to journal reports, we also researched the other web knowledge databases with unpublished clinical trials. Second, we conducted subgroup analyses to explore different SGLT-2i compounds regarding the risk-reduction effect on heart failure hospitalization, MACE, cardiovascular death, and death from any cause. Third, we used the GRADE approach to assess quality of evidence. Fourth, although three published meta-analyses have reported the discovery of SGLT-2i reducing heart failure hospitalization. Yamani et al. study (36) and Singh et al. study (37) both included only three RCTs and three other RCTs have been published after these meta-analyses published. The results of our meta-analysis were inconsistent with these three published meta-analyses but the benefit of heart failure hospitalization in type 2 diabetes patient. For instance, we found that the impact of SGLT-2i on the death from any cause have no statistical significance but SGLT-2i reduced the risk of MACE and cardiovascular death. What’s more, our result of MACE was inconsistent with Sinha et al. study that the events in the pooled analysis of the phase 3 trials reveals for MACE was statistically no significant (HR 0.83, 95% CI 0.66 to1.03, P = 0.10) (38). Therefore, we are the first using meta-analysis of randomized controlled trials who reported heart failure hospitalization, MACE, the cardiovascular death, and death from any cause in type 2 diabetes patient. We also firstly found that the impact of SGLT-2i on the death from any cause have no statistical significance which has not been reported yet.

Our study also has some limitations. There were too few clinical trials of SGLT-2i such as luseogliflozin, remogliflozin, sotagliflozin, and tofogliflozin, that can be used to meta-analyze heart failure hospitalization, MACE, the cardiovascular death, and death from any cause in type 2 diabetes patient. Without enough trials, e.g., ertugliflozin have only one article, the subgroup analysis was unable to differentiate the effects of these SGLT-2i agents.

## Conclusion

In summary, the current eight RCTs evidence showed that, SGLT-2i could reduce the risk of heart failure hospitalization, MACE, and cardiovascular death. Therefore, SGLT-2i will be an ideal choice for type 2 diabetes mellitus patient with heart failure. The impact of SGLT-2i on the death from any cause have no statistical significance; more clinical trials may provide important insights on this issue. This meta-analysis will help inform practitioners, patients, and authorities making appropriate choices in hypoglycemic therapy clinical practice.

## Data Availability Statement

The original contributions presented in the study are included in the article/**Supplementary Material**; further inquiries can be directed to the corresponding author.

## Author Contributions

YC and XZ conceptualized and designed the study. AZ, XL, HM, JK, and GQ analyzedand interpreted the data. AZ and XL drafted the manuscript. GQ modified the manuscript. AZ and XL contributed equally to this work. All authors contributed to the article and approved the submitted version.

## Funding

The author(s) received no financial support for the research, authorship, and publication of this article.

## Conflict of Interest

The authors declare that the research was conducted in the absence of any commercial or financial relationships that could be construed as a potential conflict of interest.
